# Clinical Utility of Pleural Tissue Debris Incidentally Obtained During Drainage of Pleural Effusion: A Retrospective Cohort Study

**DOI:** 10.3390/biomedicines14081726

**Published:** 2026-07-31

**Authors:** Jiwon Kim, Taewoo Kim, Han Gyeol Kim, Seung Hyeun Lee

**Affiliations:** 1 Division of Pulmonary, Allergy, and Critical Care Medicine, Department of Internal Medicine, Kyung Hee University Hospital, College of Medicine, Kyung Hee University, Seoul 02447, Republic of Korea; hi.jiwonk@gmail.com (J.K.); ktw3773@naver.com (T.K.); 2 Department of Pathology, Kyung Hee University Hospital, College of Medicine, Kyung Hee University, Seoul 02447, Republic of Korea; hangyeol.k@gmail.com; 3 Department of Precision Medicine, Graduate School, Kyung Hee University, Seoul 02447, Republic of Korea

**Keywords:** pleural tissue debris, catheter drainage, malignant pleural effusion, immunohistochemistry, molecular testing

## Abstract

**Background/Objective**: The incidence and clinical utility of pleural tissue debris, occasionally encountered in drainage systems, have not been systematically evaluated. We aimed to determine the frequency of tissue debris detection in patients undergoing drainage for pleural effusion, and its utility for the differential diagnosis of exudative effusion and pathological diagnosis of malignant pleural effusion (MPE). **Methods**: This retrospective proof-of-concept analysis included 325 consecutive patients who underwent percutaneous effusion drainage. The incidence, histopathological findings, and potential predictors of tissue debris were evaluated. For patients with MPE, the adequacy of tissue for immunohistochemistry and molecular testing was evaluated, and the diagnostic sensitivity of tissue debris for MPE was calculated and compared with that of cytology with cell blocks. **Results**: Tissue debris was detected in 151 patients (46.5%), more frequently in exudates than in transudates (51.7% vs. 23.3%, *p* < 0.001), and most frequently in patients with MPE (64.6%, *p* < 0.001). MPE was the only independent predictor of tissue debris detection (adjusted odds ratio 5.74, 95% CI 2.25–9.31). The pathological findings of the debris were classified into metastatic carcinoma/lymphoma (49.0%), acute/chronic inflammation (30.4%), fibrinoid material with inflammation (17.2%), mesothelial reaction (2.6%), and acute suppurative pleuritis/abscess (0.7%). Among the 74 cases with MPE diagnosed using tissue debris, immunohistochemistry and molecular testing were feasible in 85.1% and 69.0% cases, respectively. Diagnostic sensitivity of tissue debris for MPE was significantly higher than that of cytology (77.9% vs. 66.3%; *p* = 0.035) with a combined sensitivity of 84.2%. **Conclusions**: Systematic collection of tissue debris, a previously underrecognized clinical specimen obtained during effusion drainage, may be highly valuable for the diagnosis of MPE and adequate for immunohistochemistry and molecular testing, reducing the reliance on invasive biopsy and supporting precise oncological decisions.

## 1. Introduction

Pleural effusion is a frequently encountered clinical condition, with an estimated incidence of more than 1.5 million cases annually in the United States [1]. The initial diagnostic approach involves differentiating between transudates and exudates, typically employing Light’s criteria [2], which demonstrate high sensitivity for identifying exudates but may misclassify up to 25% of transudates, necessitating the use of the serum-effusion albumin gradient in specific cases [3].

The diagnostic challenge is often pronounced when a malignant pleural effusion (MPE) is suspected. Although various effusion biomarkers have been extensively researched, the gold standard for confirmatory diagnosis remains cytopathological detection of malignant cells [4]. However, the sensitivity of pleural fluid cytology is suboptimal (40–60%) and often requires repeat sampling or more voluminous fluid collection to improve yield [5]. Similarly, for tuberculous (TB) pleurisy, adenosine deaminase (ADA) is widely used as a robust surrogate marker; however, its specificity can be compromised in other exudative conditions, especially in empyema, and it alone cannot provide a definitive diagnosis, particularly when levels fall below the traditional cutoff of 35–40 U/L [1]. In clinical scenarios with cytology-negative suspected MPE or ADA-equivocal TB pleurisy, invasive procedures, such as closed-needle pleural biopsy or thoracoscopic biopsy, are required to obtain histological confirmation. While definitive, these procedures are inherently associated with an increased risk of complications and may not be feasible for patients of advanced age or those with severe comorbidities, highlighting the critical unmet need for a noninvasive yet accurate diagnostic tool.

Pleural tissue fragments are incidentally encountered in the drainage systems of patients undergoing catheter or chest tube drainage. Despite their potential clinical usefulness, the clinical significance of such “tissue debris” has never been reported. To address these unmet clinical needs, this study addressed the following critical clinical questions: (1) How frequently is pleural tissue debris detected in real-world clinical practice? (2) What are the distinct clinical characteristics differentiating “shedders” (patients with detectable tissue) from “non-shedders?” (3) Is this debris useful for differential diagnosis of pleural effusion? (4) In the case of MPE, is the collected debris adequate for immunohistochemical (IHC) staining and molecular testing to guide precision therapy? Through this proof-of-concept study, we aimed to establish the diagnostic utility of spontaneously exfoliated pleural tissue as a novel, noninvasive pathological resource.

## 2. Materials and Methods

### 2.1. Study Design and Subjects

This retrospective cohort study was conducted between September 2022 and December 2025 at the Kyung Hee University Hospital, a tertiary referral hospital in South Korea. We used data from the Pleural Effusion Cohort of our division, which included clinicopathological data of consecutive patients who underwent chest tube insertion or percutaneous catheter drainage for pleural effusion management. Demographic and clinical data, including age, sex, smoking status, routine blood test results, and pleural fluid analysis results, were extracted from electronic medical records. For patients diagnosed with MPE, additional pathological data were collected, including primary tumor site, histological subtype, and adequacy of IHC and/or molecular testing. The study protocol was approved by the Institutional Review Board of Kyung Hee University Hospital (No. 2025-09-030). The study was conducted in accordance with the principles of the Declaration of Helsinki.

### 2.2. Draining Procedure and Diagnostic Evaluation

All patients underwent either small-bore percutaneous catheterization or large-bore chest tube insertion. At least 50 mL of pleural fluid was collected for routine biochemical and cellular analyses. Effusions were classified using Light’s criteria and the serum-effusion albumin gradient [2,3], supplemented by conventional diagnostic criteria [1]; further details are provided in the Supplementary Materials. Notably, the diagnosis of MPE was made when cytology with cell blocks, pleural tissue debris, or pleural biopsy confirmed malignancy. In our study, pleural biopsy was performed only when the results of the former two modalities were nondiagnostic.

### 2.3. Collection and Processing of Detected Pleural Tissue Debris

According to our cohort management protocol, for better detection of tissue debris, pleural fluid collected in the chest bottle could be discarded only after thorough inspection by pulmonologists (J. Kim, T. W. Kim, and S.H. Lee); attending nurses were instructed to call these pulmonologists when they identified tissue debris in the drainage system. When present, tissue debris was collected, immediately fixed in 10% neutral-buffered formalin, and transferred to the Department of Pathology, where a qualified pathologist (H. G. Kim) performed routine pathological examinations. In cases of suspected or confirmed malignancy, pathologists performed further IHC analysis. In addition, for all cases of non-small cell lung cancer (NSCLC), as part of the routine clinical practice, we requested molecular testing, including polymerase chain reaction (PCR) or fluorescence in situ hybridization to identify genomic alterations, such as epidermal growth factor receptor (*EGFR*) mutations and anaplastic lymphoma kinase (*ALK*) rearrangements, and conducted targeted next-generation sequencing (NGS) panels.

### 2.4. Statistical Analysis

Continuous variables are expressed as medians with interquartile ranges (IQR) and categorical variables are expressed as numbers with percentages. Normality was assessed using the Shapiro–Wilk test. Comparisons among the four etiological groups (MPE, parapneumonic effusion [PPE], TB pleurisy, and transudate) were performed using the Kruskal–Wallis test with Dunn’s post hoc test and Pearson chi-square test, as appropriate. Within the MPE cohort, tissue debris and conventional diagnostic groups (cytology and pleural biopsy) were compared using the Mann–Whitney U test and chi-square or Fisher’s exact test. Tissue debris and cytology were regarded as diagnostic when the specimens were pathologically confirmed as malignant, irrespective of whether the tumor type could be assigned. Combined testing was defined according to a parallel (‘either-positive’) rule, in which a case was considered positive if cytology, tissue debris, or both were diagnostic of malignancy; combined sensitivity was calculated as the union of the two positive sets divided by the number of patients with MPE and detectable tissue debris. Pairwise comparisons of correlated sensitivities were performed using McNemar’s exact test based on discordant pairs, and the three correlated proportions were compared overall using Cochran’s Q test. Because tissue debris was not retrievable in all patients, diagnostic sensitivity was calculated in two ways: a conditional (specimen-level) sensitivity restricted to patients in whom tissue debris was obtained (n = 95), permitting paired comparison with cytology in the same individuals, and a population-level (intention-to-diagnose) sensitivity using all 147 patients with MPE as the denominator, which additionally incorporates the probability of specimen retrieval. Independent predictors of tissue debris presence were identified using multivariate logistic regression, including variables with *p* < 0.10 in the univariate analysis, with results reported as odds ratios (OR) and 95% confidence intervals (CIs). Statistical significance was set at *p* < 0.05. All analyses were performed using R software (version 4.2.0; R Foundation for Statistical Computing, Vienna, Austria).

## 3. Results

### 3.1. Baseline Characteristics of Study Subjects

**Figure 1:**
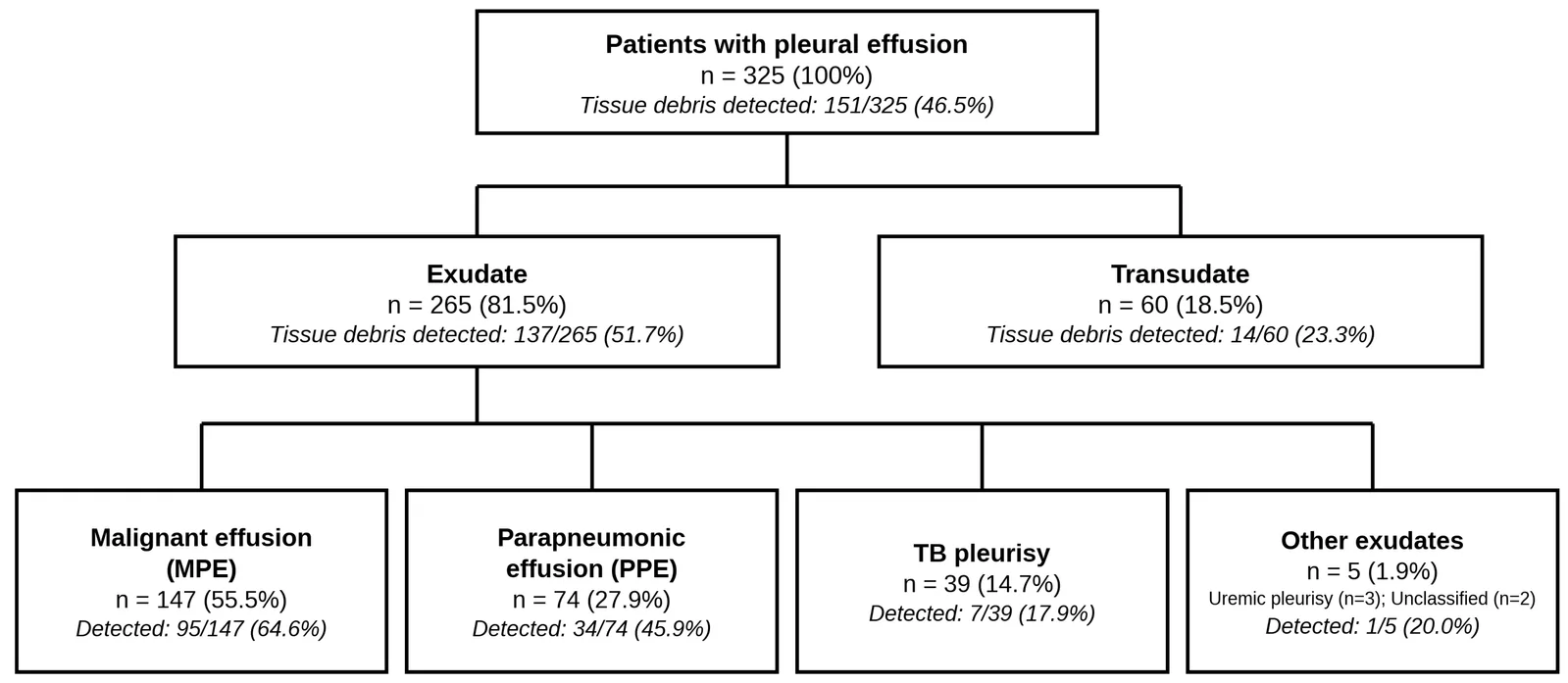
Disposition of subjects and the incidence of pleural tissue debris detection. The flowchart illustrates the distribution of 325 patients with pleural effusion based on their underlying etiologies. The number and percentage of patients who were positive for tissue debris within each group were also presented. The incidence of tissue debris was highest in those with MPE (64.6%) compared to other etiologies. MPE = malignant pleural effusion; PPE = parapneumonic effusion; TB = tuberculous.

In total, 325 patients underwent chest tube insertion or percutaneous drainage catheter placement for the diagnosis of pleural effusion during the study period. As shown in Figure 1, these comprised 60 (18.5%) with transudates and 265 (81.5%) with exudates. The exudative effusions consisted of 147 (55.5%) cases of MPEs, 74 (27.9%) PPEs, 39 (14.7%) tuberculous pleurisy, and 5 (1.9%) other exudates (3 uremic pleurisy and 2 unclassified). Baseline demographic characteristics and pleural fluid profiles according to the underlying etiology of pleural effusion are shown in Supplementary Table S1. The median age of the study population was 75.5 years; 70.4% were male. Among these, 265 (81.5%) were diagnosed with exudative pleural effusion, including 147 (55.5%) with MPE, 74 (27.9%) with PPE, and 39 (14.7%) with TB pleurisy. Transudates were diagnosed in 60 (18.5%) patients.

The transudate group was significantly older than the other groups, and male predominance was less pronounced in MPE than in the other etiologies (all *p* < 0.001). In the effusion analysis, the PPE group had the highest white blood cell count and neutrophil percentage (both *p* < 0.001), reflecting acute bacterial inflammation, whereas the TB pleurisy group showed the highest lymphocyte percentage and ADA level (both *p* < 0.001), consistent with chronic lymphocytic inflammation. As expected, based on Light’s criteria, pleural fluid protein and albumin concentrations were significantly higher in all three exudative groups (MPE, PPE, and TB pleurisy) than in the transudate group, with the highest levels observed in the TB pleurisy group (both *p* < 0.001). The pleural fluid lactate dehydrogenase level was highest in the PPE group, reflecting an intense inflammatory response (*p* < 0.001). These findings are consistent with the well-established biochemical and cellular patterns characteristic of each etiology.

### 3.2. Incidence of Pleural Tissue Debris Detection and Affecting Factors

Patient disposition and incidence of tissue debris detection are presented in Figure 1. Pleural tissue debris was detected in 151 (46.5%) patients. The incidence was significantly higher in exudates than in transudates (51.7% vs. 23.3%, *p* < 0.001). In addition, among exudates, the incidence was highest in patients with MPE (64.6%), followed by those with PPE (45.9%) and TB pleurisy (17.9%).

Univariate and multivariate logistic regression analyses were performed to identify factors associated with tissue debris shedding (Table 1). In the univariate analysis, age, sex, smoking history, history of previous drainage procedures within 6 months, and types of drainage procedures did not differ between shedders and non-shedders. MPE was associated with more frequent detection of tissue debris (*p* < 0.001). In the multivariate analysis, diagnosis of MPE was the only independent predictor of the detection of tissue debris (adjusted OR 5.74, 95% CI 2.25–9.31; *p* < 0.001).

### 3.3. Pathological Classification of Tissue Debris and Its Distribution According to Different Etiologies

**Figure 2:**
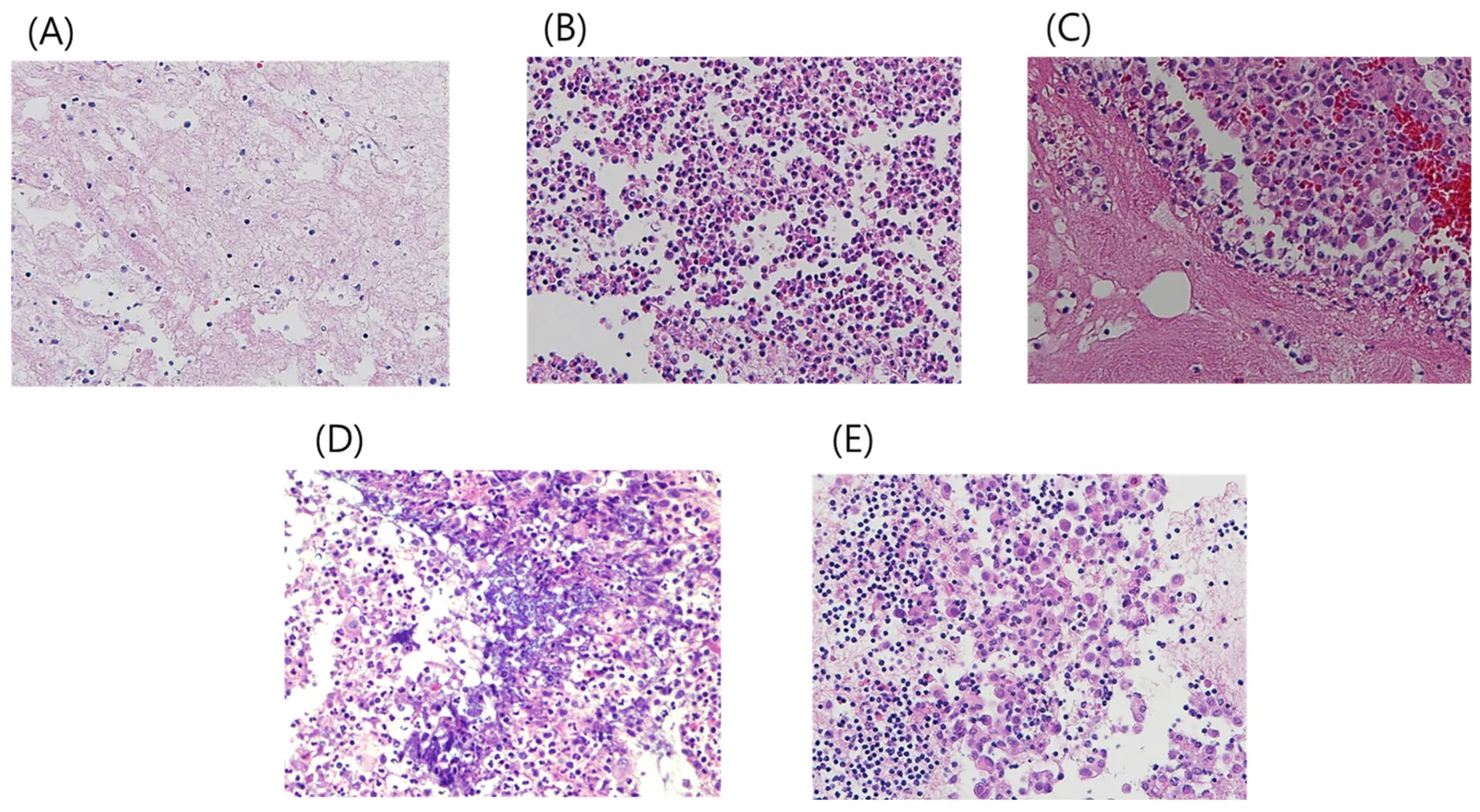
Five histopathological classifications of pleural tissue debris. Fibrinoid material with inflammatory cells (**A**), acute or chronic inflammation (**B**), mesothelial reactions (**C**), acute suppurative pleuritis or abscess (**D**), and metastatic carcinoma or lymphoma (**E**). (H&E stain, ×400).

Although tissue debris is occasionally encountered in clinical practice, its histopathological classification has not previously been reported. As shown in Figure 2, the tissue debris exhibited a wide spectrum of diagnostically informative findings. Some cases involved sparse inflammatory cells embedded in fibrinoid material or dense mixed acute and chronic infiltration, and reactive mesothelial proliferation was observed in some cases. Necrotic cellular debris with bacterial aggregates was identified in cases of infectious etiology. Importantly, many cases exhibited atypical cells with prominent nucleoli and irregular nuclear contours mixed with inflammatory cells, consistent with metastatic carcinoma. Based on the dominant histopathological features, we classified the pleural tissue debris into five categories (Figure 2A–E): (1) fibrinoid material with inflammatory cells, (2) acute or chronic inflammation, (3) mesothelial reactions, (4) acute suppurative pleuritis or abscess, and (5) metastatic carcinoma or lymphoma.

The distribution of these pathological findings by etiology is summarized in Table 2. Among the 151 patients with detectable tissue debris, the most common histopathological finding was metastatic carcinoma or lymphoma (n = 74, 49.0%), followed by acute or chronic inflammation (n = 46, 30.4%), fibrinoid material with inflammatory cells (n = 26, 17.2%), mesothelial reaction (n = 4, 2.6%), and acute suppurative pleuritis or abscess (n = 1, 0.7%).

The distribution of histopathological findings differed significantly among the three exudative etiologies. Notably, metastatic carcinoma or lymphoma was identified exclusively in the MPE group (*p* < 0.001), whereas the remaining MPE shedders showed acute or chronic inflammation (10.5%) or fibrinoid material with inflammatory cells (10.5%). In contrast, acute or chronic inflammation predominated in the infectious etiologies, accounting for 67.6% of PPE shedders and 57.1% of TB pleurisy shedders, compared with only 10.5% of MPE shedders (*p* < 0.001). Fibrinoid material with inflammatory cells showed a borderline difference among the three groups (10.5%, 26.5%, and 28.6% in MPE, PPE, and TB pleurisy, respectively; *p* = 0.066), suggesting a nonspecific inflammatory pattern shared across etiologies. Mesothelial reactions and acute suppurative pleuritis or abscesses were observed sporadically and did not differ significantly between the groups. Granulomatous inflammation, the histopathological hallmark of TB pleurisy, was not identified in any of the seven patients with TB pleurisy who shed tissue debris. Among the 14 transudate shedders, acute or chronic inflammation (64.3%) and fibrinoid material with inflammatory cells (28.6%) were the predominant histological findings.

### 3.4. Demographics and Cancer Types in MPE Patients

Table 3 summarizes the tumor types in the patients with MPE (n = 147); 136 patients (92.5%) were diagnosed with intrathoracic cancer and 11 (7.5%) with extrathoracic cancer. Lung cancer was the most prevalent (n = 130), followed by pleural mesothelioma (n = 6), breast cancer (n = 5), ovarian cancer (n = 3), lymphoma (n = 2), and gastric cancer (n = 1). Among the lung cancers, lung adenocarcinoma (LUAD, n = 96) was the most frequent, followed by lung squamous cell carcinoma (n = 20) and SCLC (n = 14).

To identify potential differences in the shedding rate according to cancer type, we compared cancer distribution between two groups: patients diagnosed using tissue debris (n = 74) and those diagnosed by conventional methods, including cytology or pleural biopsy (n = 78). Age and sex were comparable between the two groups. The overall distribution of malignancy types differed marginally between the groups (*p* = 0.044), driven primarily by the presence of five “unclassified malignancy” cases in the tissue debris group. The five cases in the tissue debris group were initially diagnosed only as “metastatic carcinoma,” thereby requiring further pleural biopsy for confirmatory diagnosis, which subsequently revealed the following: lymphoma (n = 1), mesothelioma (n = 1), ovarian cancer (n = 1), and small cell lung cancer (n = 2).

When restricted to cases with assigned cancer types, the proportions of intrathoracic and extrathoracic malignancies were similar between the two groups, and the distribution of intrathoracic subtypes did not differ significantly. Notably, SCLC occurred less frequently in the tissue debris group than in the conventional group (5.4% vs. 12.8%, *p* = 0.159), although the difference was not statistically significant.

We further analyzed the tissue adequacy for IHC or molecular testing of tissue debris. A list of IHC markers for various tumors is presented in Supplementary Table S2. IHC was possible in 63 (85.1%) of 74 patients whose diagnoses were made using tissue debris. The adequacy of molecular testing, including PCR and NGS, was assessed in tissue debris-positive patients with NSCLC, and testing was possible in 40/58 (69.0%) patients. The adequacy of tissue debris for molecular testing slightly differed by platform, being numerically higher for PCR-based assays (69.0%) than for targeted NGS panels (60.3%), and every specimen sufficient for NGS was also sufficient for PCR (Supplementary Table S5). This gradient is clinically relevant, as it suggests that tissue debris can reliably support rapid single-gene testing for actionable alterations, whereas comprehensive genomic profiling may require a larger retrieved fragment or a complementary specimen.

### 3.5. Sensitivity of Cytology and Tissue Debris for Diagnosis of MPE

**Figure 3:**
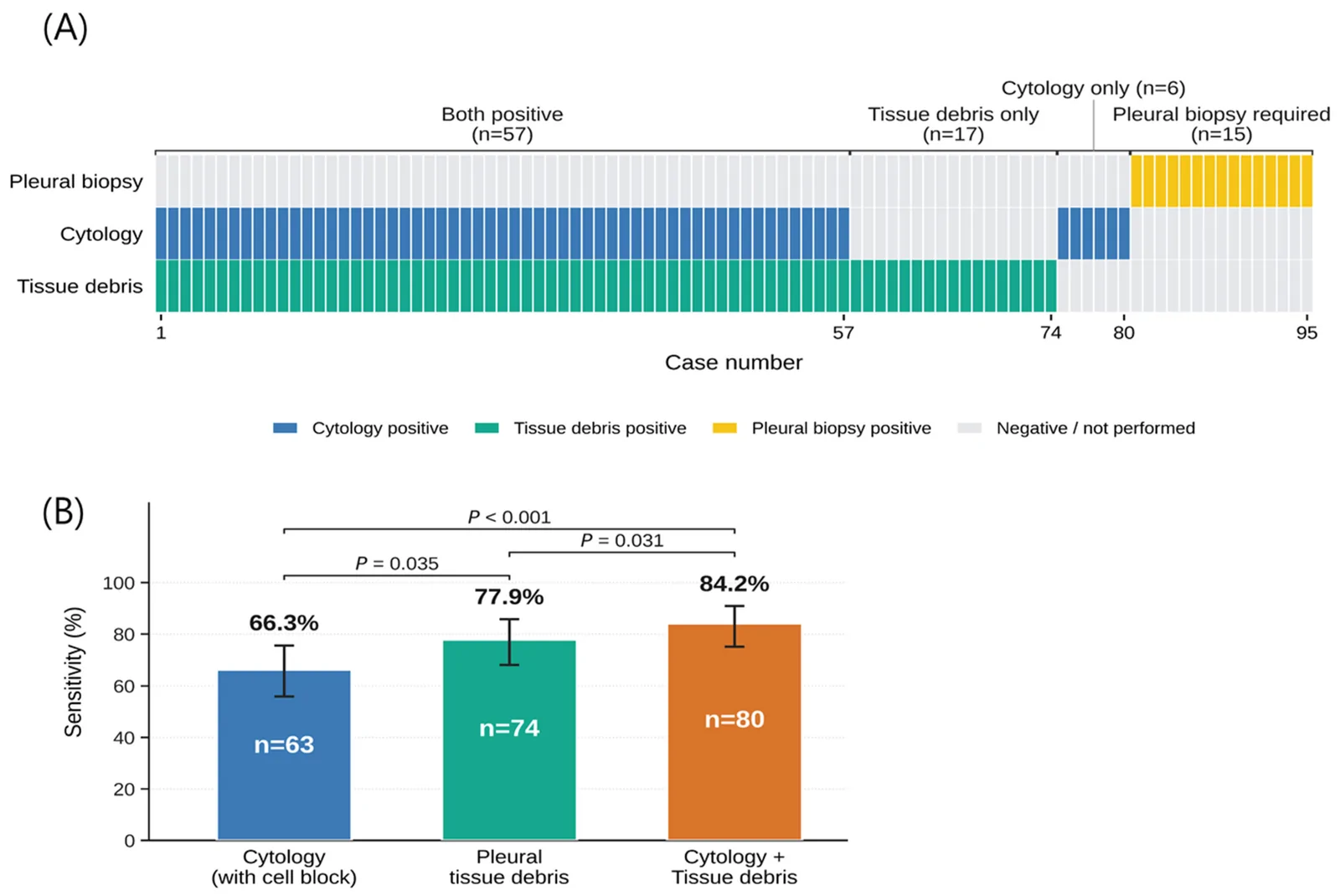
Comparison of diagnostic performance between cytology and tissue debris in patients with MPE who had detectable tissue debris (n = 95). (**A**) Patient-level distribution of diagnostic results. Each vertical column represents one patient. Among 95 patients, 57 were positive by both modalities, while tissue debris identified 17 additional cases and cytology identified six additional cases. The remaining 15 patients required a pleural biopsy for the definitive diagnosis. (**B**) Bar graph showing the diagnostic sensitivity of cytology, pleural tissue debris, and their combination for the diagnosis of MPE. Error bars indicate 95% confidence intervals calculated by the exact method. Numbers within each bar represent the number of cases identified by each modality. MPE = malignant pleural effusion.

To compare diagnostic performance, we calculated the sensitivity of cytology and tissue debris in patients with MPE who had detectable tissue debris (n = 95). Figure 3A summarizes the patient-level distribution of diagnostic results. Tissue debris was confirmed as MPE in 74 cases, with confirmation by cytology in 63 cases, and 57 cases identified using both modalities. Notably, tissue debris was detected in 17 additional cases overlooked by cytology, whereas cytology identified six additional cases overlooked by tissue debris. Pleural biopsies were performed in 15 patients who were not diagnosed using either modality. The detailed conditional (specimen-level) diagnostic sensitivities and paired comparisons are summarized in Supplementary Tables S3 and S4. The sensitivity of tissue debris detection (77.9%) was significantly higher than that of cytology (66.3%; *p* = 0.035). The combined sensitivity reached 84.2%, significantly exceeding that of either modality alone (combined vs. cytology, *p* < 0.001; combined vs. tissue debris, *p* = 0.031; Figure 3B).

Additionally, we also calculated the population-level sensitivities for the entire MPE cohort (n = 147, Supplementary Table S3). Among the 52 patients with MPE in whom tissue debris was not retrieved, 40 were diagnosed by cytology with cell blocks and 12 required pleural biopsy. As expected, the sensitivity of tissue debris alone decreased to 50.3% (74/147) because specimen retrieval was possible in only 64.6% of patients with MPE. The sensitivity of cytology with cell block was 70.1% (103/147). The combined diagnostic sensitivity for MPE remained high at 81.6% (120/147) and was significantly superior to either modality alone (both *p* < 0.001), which is consistent with the conditional sensitivity analysis.

### 3.6. Representative Clinical Case

**Figure 4:**
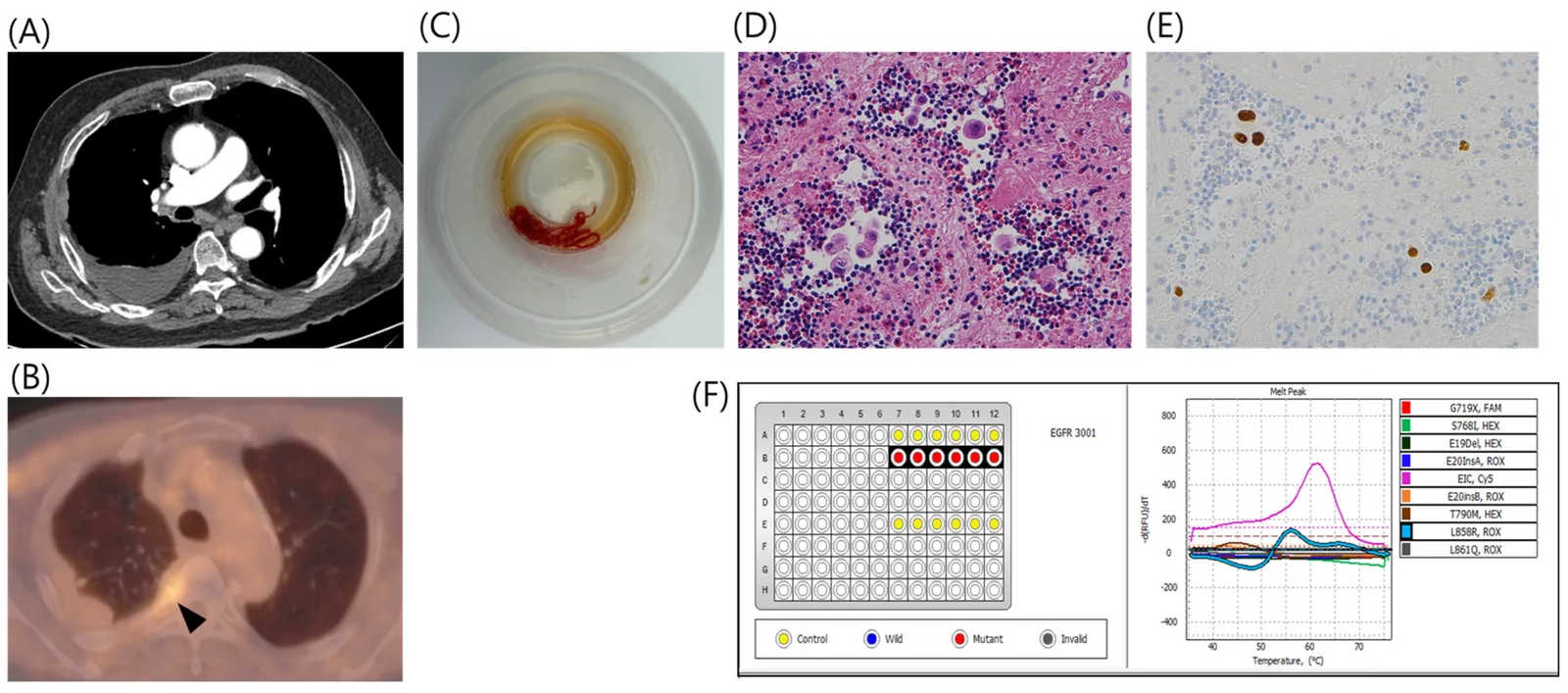
Clinical and pathological findings of a representative case. (**A**) Contrast-enhanced chest CT scan showing a large right-sided pleural effusion with multiple pleural nodules. (**B**) PET/CT scan revealing hypermetabolism in the right upper pleura (arrowhead), suggestive of pleural metastasis. (**C**) Gross appearance of the reddish, thread-like tissue debris collected from the drainage extension tube. (**D**) Microscopic findings of the tissue debris showing nests of adenocarcinoma cells within a fibrinous background (H&E stain, ×400). (**E**) Immunohistochemical staining for TTF-1 showing strong nuclear expression in the tumor cells (×400), consistent with lung adenocarcinoma. (**F**) EGFR mutation analysis using the PANA Mutyper™ PCR assay. The melting peak analysis demonstrates a distinct peak for the L858R mutation (cyan line) alongside the internal control (pink line), confirming the presence of the EGFR L858R mutation.

A 68-year-old man with a 30-pack-year smoking history presented with dyspnea that had persisted for 3 months. He had recently undergone percutaneous coronary angioplasty of the right coronary artery and received dual antiplatelet therapy (DAPT) with aspirin and clopidogrel. Chest imaging revealed a right-sided pleural effusion with pleural nodules (Figure 4A); ^18^F-FDG PET demonstrated hypermetabolic pleural thickening suggestive of MPE (Figure 4B, arrowhead). Pleural fluid analysis revealed a lymphocyte-dominant exudate with a low ADA (13.5 IU/L); two consecutive cytology tests with cell blocks were negative. Because an invasive biopsy was contraindicated by the risk of stent thrombosis associated with DAPT interruption, no further procedure was performed. On hospital day 3, thread-like tissue debris (7–8 cm) was unexpectedly identified in the transparent extension tube (Figure 4C) and subjected to pathological evaluation. Histology revealed an adenocarcinoma with strong TTF1 positivity (Figure 4D, E), which was consistent with LUAD. Subsequent molecular testing revealed an *EGFR* L858R mutation (PANA Mutyper™, PANAGENE; Figure 4F), and first-line osimertinib was initiated for stage IVA disease.

## 4. Discussion

To our knowledge, this is the first study to systematically evaluate the incidence and clinical utility of pleural tissue debris obtained incidentally during routine drainage. The frequency of pleural tissue debris detection was 46.5% in the overall population, with more frequent detection in exudates than in transudates (51.7% vs. 23.3%). MPE was the only independent predictor of tissue debris detection. Among MPE shedders, various intrathoracic and extrathoracic cancer types were confirmed using tissue debris, and most samples were adequate for IHC or molecular testing. The diagnostic sensitivity of tissue debris for MPE was 77.9%, which was significantly higher than that of cytology using cell blocks (66.3%; *p* = 0.035), and the combined sensitivity of these two modalities was 84.2%. These findings suggest that this previously under-recognized specimen may serve as a valuable adjunct for the differential diagnosis of pleural effusion, particularly in patients with suspected MPE.

One of the most notable findings of this study is that pleural tissue debris was detected considerably more frequently than previously anticipated. It occurred in approximately half of the screened patients and was more prevalent in exudative effusions than in transudates. Although the mechanism underlying spontaneous shedding of pleural tissue in patients with pleural effusion remains unclear, we propose several hypotheses for this phenomenon. First, a procedure-related mechanism may be responsible, wherein the insertion of a catheter or chest tube causes mechanical injury to the pleura, leading to the dislodgement of tissue fragments. Second, the higher frequency of exudates may be attributed to pleural inflammation, which potentially facilitates the separation of small pleural segments from the pleural space. However, procedural injury and pleural inflammation do not fully account for the high frequency of MPE. This unique association suggests a third cancer-specific hypothesis that may be intrinsic to the biological nature of malignancies. Malignant invasion renders the pleural surface more friable, leading to both spontaneous exfoliation and increased susceptibility to mechanical dislodgement during procedures [6,7]. This hypothesis is further supported by evidence that cancer tissues frequently exhibit weakened structural integrity due to loosened cell-to-cell junctions, often characterized by the downregulation of E-cadherin, which facilitates the detachment of cellular clusters from the primary tumor site [8,9]. Further mechanistic studies are warranted to elucidate the biological processes underlying spontaneous pleural tissue shedding.

Despite being the diagnostic cornerstone of MPE, pleural fluid cytology has limitations that remain unresolved. A negative cytology result does not exclude malignancy and frequently necessitates additional invasive procedures, leading to diagnostic delay, increased patient burden, and higher healthcare costs [10,11]. The diagnostic yield from repeat sampling diminishes rapidly, with the sensitivity of the second and third samples falling to approximately 22% and 14%, respectively [11]. Cytologic sensitivity is particularly poor in mesothelioma and lung squamous cell carcinoma, in which exfoliation of malignant cells is minimal and architectural evaluation, such as assessment of chest wall invasion, is not feasible on dispersed cells [5]. Furthermore, even when cellularity is adequate, results categorized as “suspicious” or “atypical” lack diagnostic certainty and often fail to support definitive oncologic decision-making. Previous large-scale studies have reported that the diagnostic sensitivity of pleural fluid cytology, even when combined with cell blocks, ranges from 46% to 60% [5,10,12,13]. A recent meta-analysis involving 36 studies and 6057 patients reported a pooled sensitivity of 58.2% (95% CI, 52.5–63.9%), with substantial heterogeneity across cancer types: LUAD showed the highest sensitivity (83.6%), whereas lung squamous cell carcinoma (24.2%) and mesothelioma (28.9%) showed markedly lower yields [14]. The diagnostic sensitivity of cytology observed in our cohort (66.3%) was consistent with that of these reports. Notably, tissue debris achieved a diagnostic sensitivity of 77.9% in our cohort, significantly outperforming cytology and additionally identified 17 cases (23.0%) not detected by cytology. The combined sensitivity reached 84.2%, suggesting that integration of tissue debris analysis with conventional cytology could substantially reduce the need for additional invasive procedures such as pleural biopsy and medical thoracoscopy for the definitive diagnosis of MPE.

Of note, this combined sensitivity remains lower than that of medical thoracoscopy, for which pooled sensitivities of 92–93% have been reported [15], and tissue debris is therefore not proposed as an alternative to thoracoscopic biopsy. The two approaches are nonetheless not directly comparable, as tissue debris entails no additional procedure, anesthesia, or risk, whereas thoracoscopy carries a pooled complication rate of approximately 4% [15]. Given its high specificity and negligible marginal cost, tissue debris is best positioned as a routine adjunct that precedes invasive investigation, with thoracoscopy reserved for patients in whom both cytology and tissue debris are non-diagnostic—18.4% of the present cohort.

Another important observation was the adequacy of pleural tissue debris for additional pathological examination. Biomarker testing including IHC for predictive markers (e.g., programmed death-ligand 1 [PD-L1]) and molecular profiling for actionable driver alterations is essential for decision-making [16]. According to current guidelines, all cytology preparations, including direct smears, liquid-based preparations, and cell blocks, may be used for ancillary studies if cellularity and preservation are adequate [17]. In our cohort, IHC was successfully performed in 85.1% of MPE shedders and molecular testing in 69.0% of NSCLC shedders, enabling identification of actionable mutations and initiation of targeted therapy, as evident in the aforementioned case. The IHC adequacy of tissue debris (85.1%) was comparable to that reported in a previous study, in which effusion cytology with cell blocks was adequate for PD-L1 IHC in 92% of NSCLC cases [17]. In contrast, the adequacy for molecular testing in our cohort (69.0%) was lower than that reported in another study, in which NGS using pleural fluid of LUAD patients achieved an adequacy of 92% [18]. Theoretically, tissue debris includes macroscopic, three-dimensional fragments of tumors rather than dispersed cellular aggregates; thus, it may provide a specimen with adequate quantity and quality of cells, similar to that obtained using standard pleural biopsy. Although large-scale studies are warranted to validate our findings, the adequacy of IHC and molecular testing observed in this study suggests that the combination of tissue debris and cytology not only reduces the need for invasive procedures, such as pleural biopsy, but also facilitates comprehensive assays required for both definitive diagnosis and therapeutic decision-making.

In parallel with the development of blood-based NGS, liquid biopsy using pleural effusion has become an area of active investigation [19]. In a recent prospective study of 50 patients with newly diagnosed advanced NSCLC with malignant effusion, actionable mutations were identified in 88% of patients by NGS of effusion cell-free DNA (cfDNA) in effusion, which was significantly higher than the rates obtained using the cell pellet (78%) or tumor tissue (66%) (*p* = 0.031) [20]. Beyond genotyping, cfDNA methylation profiling of pleural fluid has been evaluated as a diagnostic marker for distinguishing malignant from benign effusions [21]. Nevertheless, cfDNA-based NGS has inherent limitations. It provides genotype without morphology and therefore cannot establish a pathological diagnosis, assign tumor lineage, or identify histological transformation such as small cell transformation after targeted treatment, all of which require intact tissue. It also cannot support protein-level assessment by IHC, including PD-L1 expression, which remains essential for selecting immunotherapy. Tissue debris, in contrast, provides intact tissue amenable to histological evaluation, IHC, and molecular testing without additional invasive procedure. The combination of tissue debris and effusion cfDNA may therefore be complementary, and further studies are warranted to define the optimal integration of these two specimens.

The strengths of this study include its relatively large sample size, inclusion of consecutive patients to minimize selection bias, and the systematic histopathological, immunohistochemical, and molecular characterization of all retrieved tissue debris specimens. Of note, the reported incidence of 46.5% may be a reflection of an institutional protocol rather than routine clinical practice, and thus the true incidence in centers without such a protocol may be substantially lower. However, our protocol requires no additional equipment, no additional cost, and no invasive manipulation, consisting solely of visual inspection of fluid that would otherwise be discarded. It can thus be readily reproduced across centers in validation studies, minimizing between-center heterogeneity in specimen ascertainment. Moreover, given that the retrieved debris yielded a definitive diagnosis in a substantial proportion of patients with MPE and was adequate for IHC and molecular characterization, we consider that systematic inspection of the drainage system deserves consideration as a standard component of the diagnostic work-up in patients undergoing drainage for pleural effusion.

This study had some limitations. First, this was a retrospective, single-center study, which may have introduced an inherent selection bias. Although we included consecutive patients to minimize bias, our cohort predominantly consisted of patients with intrathoracic malignancies. Therefore, the findings may not be fully generalizable to MPE caused by other cancers originating from the extrathoracic organs. Second, because the collection of tissue debris was based on incidental discovery during routine drainage, no standardized protocol exists for its identification and retrieval. The frequency of tissue debris may have been underestimated if physicians and nursing staff had not consistently monitored the drainage system. Third, although the detection rate of tissue debris did not differ significantly between small-bore catheters and large-bore chest tubes in the present study, we were unable to assess whether the size or amount (weight) of the retrieved debris differed according to drainage caliber. Fourth, we were unable to identify disease-specific histopathological features that could distinguish PPE from TB pleurisy on the basis of tissue debris alone. Granulomas were not identified in any case of TB pleurisy in our study, possibly reflecting the relatively small number of TB cases. In addition, fragment size, rather than fixation, could limit granuloma detection, since all specimens were immediately formalin-fixed and paraffin-embedded. Of note, previous studies have shown that granuloma macrophages undergo epithelialization with upregulation of E-cadherin and junctional proteins and are encased by a fibrotic cuff, a state opposed to the loss of cell–cell adhesion observed in malignancies; granulomatous tissue would therefore be expected to resist spontaneous exfoliation [22,23]. Accordingly, ancillary mycobacterial staining and nucleic acid amplification testing of retrieved debris may be required to establish its diagnostic value in this setting. Fifth, the histopathological classification of pleural tissue debris proposed in this study should be considered preliminary rather than definitive. Although the five categories were derived from the dominant histopathological features observed in 151 shedders, the sample size remained modest, and the framework requires refinement and expansion as additional cases accumulate in future studies. Finally, although our findings provide strong proof-of-concept, external validation in larger multicenter cohorts is essential to confirm the reproducibility and generalizability of our findings. To overcome these limitations and validate our findings, a prospective multicenter study with standardized collection and handling protocols is underway. Confirmation of the present findings would establish pleural tissue debris as a routine diagnostic specimen for both the pathological diagnosis and comprehensive genomic profiling of patients with MPE.

## 5. Conclusions

Historically, diagnostic evaluation of pleural effusions has relied predominantly on biochemical and molecular analyses of liquid components. This study introduced a novel and potentially practical approach that utilizes incidentally detected pleural tissue debris. This previously under-recognized specimen is frequently detected, particularly in MPE, and serves as a highly valuable pathological resource. The combined high sensitivity of tissue debris and conventional cytology for the diagnosis of MPE and the relatively high adequacy of ancillary pathologic testing could reduce the clinical necessity for invasive procedures and enable molecular characterization and confirmatory diagnosis. Although our data warrant validation in future investigations, these tissue fragments may offer a practical, minimally invasive, and effective strategy for precision oncology-based decision making.

## Figures and Tables

**Table 1:** Univariate and multivariate logistic regression analyses of factors associated with pleural tissue debris shedding (N = 325).

Variable	All (N = 325)	Shedder (n = 151)	Non-Shedder (n = 174)	Univariate Analysis		Multivariate Analysis	
				OR (95% CI)	*p* Value *	aOR (95% CI)	*p* Value
Age (years)							
<70	100 (30.8)	49 (32.5)	51 (29.3)	Reference	—	—	—
≥70	225 (69.2)	102 (67.5)	123 (70.7)	0.86 (0.54–1.38)	0.623	—	—
Sex							
Male	229 (70.5)	105 (69.5)	124 (71.3)	Reference	—	—	—
Female	96 (29.5)	46 (30.5)	50 (28.7)	1.09 (0.67–1.75)	0.827	—	—
Smoking history							
Never smoker	110 (33.8)	50 (33.1)	60 (34.5)	Reference	—	—	—
Ever smoker	215 (66.2)	101 (66.9)	114 (65.5)	1.06 (0.67–1.69)	0.886	—	—
History of previous PCD within 6 months							
No	283 (87.1)	133 (88.1)	150 (86.2)	Reference	—	—	—
Yes	42 (12.9)	18 (11.9)	24 (13.8)	0.85 (0.44–1.63)	0.737	—	—
Type of draining procedure							
Drainage catheter (10 to 12-Fr)	298 (91.7)	136 (90.1)	162 (93.1)	Reference	—	—	—
Chest tube (24-Fr)	27 (8.3)	15 (9.9)	12 (6.9)	1.49 (0.67–3.29)	0.431	—	—
Etiology of pleural effusion							
Transudate	60 (18.5)	14 (9.3)	46 (26.4)	Reference	—	Reference	—
Non-malignant exudate	118 (36.3)	42 (27.8)	76 (43.7)	1.82 (0.90–3.68)	0.098	1.08 (0.51–2.44)	0.774
MPE	147 (45.2)	95 (62.9)	52 (29.9)	6.00 (3.02–11.93)	<0.001	5.74 (2.25–9.31)	<0.001

Data are presented as n (%). aOR, adjusted odds ratio; CI, confidence interval; MPE, malignant pleural effusion; OR, odds ratio; PCD, percutaneous catheter drainage. * *p*-values for between-group comparisons (shedder vs. non-shedder) were calculated using the Pearson chi-square test, and Fisher’s exact test was applied when expected cell counts were less than 5.

**Table 2:** Distribution of histopathologic findings of pleural tissue debris according to the etiology of pleural effusion (n = 151 shedders).

		Exudate (n = 137)				*p* Value *	Transudate (n = 14)
Pathologic Finding	All (n = 151)	MPE (n = 95)	PPE (n = 34)	TB Pleurisy (n = 7)	Other Exudate (n = 1)		
Metastatic carcinoma/lymphoma	74 (49.0)	74 (77.9)	0 (0)	0 (0)	0 (0)	<0.001	0 (0)
Acute/Chronic inflammation	46 (30.4)	10 (10.5)	23 (67.6)	4 (57.1)	0 (0)	<0.001	9 (64.3)
Fibrinoid material & inflammation	26 (17.2)	10 (10.5)	9 (26.5)	2 (28.6)	1 (100)	0.066	4 (28.6)
Mesothelial reaction	4 (2.6)	1 (1.1)	1 (2.9)	1 (14.3)	0 (0)	0.165	1 (7.1)
Acute suppurative pleuritis/abscess	1 (0.7)	0 (0)	1 (2.9)	0 (0)	0 (0)	0.306	0 (0)

Data are presented as n (%). Percentages represent proportions within each etiological group. MPE, malignant pleural effusion; PPE, parapneumonic effusion; TB, tuberculosis. * *p* values were calculated to compare histopathological findings among the three exudative etiologies (MPE, PPE, and TB pleurisy; total n = 136). The “Other exudate” (n = 1) and “Transudate” (n = 14) groups were excluded from statistical comparison.

**Table 3:** Demographics and distribution of malignancy types according to the diagnostic method of malignant pleural effusion (n = 147).

		Cytopathologic Confirmation		
Variable	All MPE (n = 147)	Tissue Debris (n = 74)	Conventional (Cytology or Pleural Biopsy, n = 78) *	*p* Value
Age, years	73.0 (62–81)	73.5 (61–80)	72.5 (64–82)	0.684
Male sex	96 (65.3)	46 (62.2)	50 (64.1)	0.937
Types of malignancy				0.044
Intrathoracic	136 (92.5)	65 (87.8)	71 (91.0)	0.542
Lung adenocarcinoma	96 (65.3)	48 (64.9)	48 (61.5)	0.702
Lung squamous cell carcinoma	20 (13.6)	10 (13.5)	8 (10.3)	0.615
Small cell lung cancer	14 (9.5)	4 (5.4)	10 (12.8)	0.159
Mesothelioma	6 (4.1)	3 (4.1)	3 (3.8)	1.000
Extrathoracic	11 (7.5)	4 (5.4)	7 (9.0)	1.000 †
Breast cancer	5 (3.4)	2 (2.7)	3 (3.8)	
Ovarian cancer	3 (2.0)	1 (1.4)	2 (2.6)	
Gastric cancer	1 (0.7)	0 (0)	1 (1.3)	
Lymphoma	2 (1.4)	1 (1.4)	1 (1.3)	
Unclassified malignancy	0 (0)	5 (6.8)	0 (0) ‡	—

Data are presented as median (interquartile range) for age and n (%) for categorical variables. * Included five patients whose diagnoses were non-confirmatory using tissue debris and thus underwent further pleural biopsy. † *p* values were not calculated for individual extrathoracic subtypes because of expected cell counts below five; extrathoracic malignancies were compared as a single group. ‡ A definitive pathological diagnosis was established in all patients in the conventional group.

## Data Availability

The original data generated and analyzed in this study are included in the article and its Supplementary Materials. Please contact the corresponding author for further inquiry.

## Supplementary Materials

The following supporting information can be downloaded at: https://www.mdpi.com/article/10.3390/biomedicines14081726/s1, Supplementary Methods: Draining Procedure and Diagnostic Evaluation; Supplementary Table S1: Baseline characteristics of the overall study population (N = 325); Supplementary Table S2: Markers for immunohistochemistry according to various tumors; Supplementary Table S3: Conditional and population-level diagnostic sensitivity of cytology, pleural tissue debris, and their combination for the diagnosis of malignant pleural effusion; Supplementary Table S4: Statistical comparison of paired diagnostic sensitivities (McNemar’s test and Cochran’s Q test); Supplementary Table S5: Adequacy of pleural tissue debris for molecular testing according to assay platform in patients with non-small cell lung cancer (n = 58).

## Abbreviations

The following abbreviations are used in this manuscript:

ADA	Adenosine deaminase
aOR	Adjusted odds ratio
CI	Confidence interval
DAPT	Dual antiplatelet therapy
EGFR	Epidermal growth factor receptor
IHC	Immunohistochemical
LUAD	Lung adenocarcinoma
MPE	Malignant pleural effusion
NGS	Next-generation sequencing
NSCLC	Non-small cell lung cancer
OR	Odds ratio
PCR	Polymerase chain reaction
PD-L1	Programmed death-ligand 1
PPE	Parapneumonic effusion
TB	Tuberculous
