# Supplementary material for: Clinical Utility of Pleural Tissue Debris Incidentally Obtained During Drainage of Pleural Effusion: A Retrospective Cohort Study
Source: Biomedicines. 2026 Jul 31;14(8):1726. doi: 10.3390/biomedicines14081726 (PMC13509878; doi:10.3390/biomedicines14081726)
Supplement: Supplementary file 1 [file biomedicines-14-01726-s001.zip › biomedicines-4446257-supplementary.pdf]

Supplementary Material

Clinical Utility of Pleural Tissue Debris Incidentally Obtained During Drainage of Pleural Effusion: A Retrospective Cohort Study

Jiwon Kim [1], Taewoo Kim [1], Han Gyeol Kim [2] and Seung Hyeun Lee [1,3,*]

[1] Division of Pulmonary, Allergy, and Critical Care Medicine, Department of Internal Medicine, Kyung Hee University Hospital, College of Medicine, Kyung Hee University, Seoul 02447, Republic of Korea
[2] Department of Pathology, Kyung Hee University Hospital, College of Medicine, Kyung Hee University, Seoul 02447, Republic of Korea
[3] Department of Precision Medicine, Graduate School, Kyung Hee University, Seoul 02447, Republic of Korea
* Correspondence: humanmd04@hanmail.net; Tel.: +82-2-958-8511

METHODS

Draining Procedure and Diagnostic Evaluation

All patients underwent either small-bore percutaneous catheterization or large-bore chest tube insertion under local anesthesia via infiltration of 1% lidocaine. Drainage procedures were performed under ultrasound guidance by trained interventional radiologists using either a 10.2-Fr to 12-Fr pigtail-locking catheter (Multi-purpose Drainage Catheter II™; Sungwon Medical, Cheongju, Republic of Korea) or a 10-Fr J-tip catheter (Flexima™ APD™; Boston Scientific, Marlborough, MA, USA) via the Seldinger technique. Standard chest tubes (24-Fr; Dongwha Panda Co., Ltd., Incheon, Republic of Korea) were placed by thoracic surgeons using the blunt dissection technique. Following insertion, catheters and tubes were connected to a 1,000-mL transparent drainage system (CB (L-1000); Dongwha Panda Co., Ltd.) to maintain vacuum and facilitate the evacuation of air or pleural fluid.

A minimum of 50 mL of pleural effusion was collected for routine analysis, which included: (1) gross appearance; (2) total and differential cell counts; (3) biochemical measurement of pH, protein, albumin, amylase, glucose, lactate dehydrogenase (LDH),

and adenosine deaminase (ADA) levels; (4) cytology with cell block preparation (performed at least twice); and (5) microbiological examination, including Gram staining, acid-fast bacillus (AFB) staining, tuberculosis and bacterial cultures, and polymerase chain reaction (PCR) for Mycobacterium tuberculosis and non-tuberculous mycobacteria (NTM). Effusions were classified as exudative or transudative according to Light's criteria, using pleural fluid and serum protein and LDH levels.[1] A serum–pleural fluid protein gradient>3.1 g/dL or a serum–pleural fluid albumin gradient>1.2 g/dL was used to reclassify effusion as transudative.[2]

Parapneumonic effusion (PPE) was defined as a neutrophil-dominant exudate along with the presence of pulmonary infection, in which the patient had fever, purulent sputum, and pneumonic infiltration on chest radiography in response to antibiotic treatment. TB pleurisy was diagnosed in cases of: lymphocyte dominance and high ADA levels (>40 IU/L) in the pleural fluid, pathological confirmation of caseating granuloma in a pleural biopsy specimen, or positive TB culture or TB PCR using pleural fluid. MPE was diagnosed when cytopathological confirmation of malignant cells was obtained using cytology with cell blocks, tissue debris, or a pleural biopsy. Pleural biopsies were not performed in cases in which a definitive pathological diagnosis was made using cytology or pleural tissue debris.

References

1. Heffner JE, Brown LK, Barbieri CA. Diagnostic value of tests that discriminate between exudative and transudative pleural effusions. Primary Study Investigators. *Chest.* 1997;111(4):970-980.
2. Bielsa S, Porcel JM, Castellote J, Mas E, Esquerda A, Light RW. Solving the Light's criteria misclassification rate of cardiac and hepatic transudates. *Respirology.* 2012;17(4):721-726.

Supplementary Table S1. Baseline characteristics of the overall study population (N=325)

Variable	Total (N=325)	Exudate (n=265)				Transudate (n=60)	*p value**
		MPE (n=147)	PPE (n=74)	TB pleurisy (n=39)	Others (n=5)		
**Age (years)**	75.5 (69.0–84.0)	72.5 (67.0–82.0)	75.8 (72.0–82.0)	73.0 (66.0–86.0)	76.1 (69.0–85.0)	80.4 (73.5–86.5)	< 0.001
**Sex, male, n (%)**							0.109
Male	229 (70.4)	90 (61.2)	57 (77.0)	28 (71.8)	4 (80.0)	47 (78.3)	
Female	96 (29.6)	57 (38.8)	17 (23.0)	11 (28.2)	1 (20.0)	13 (21.7)	
**Smoking status**							0.055
Never smoker	110 (33.8)	50 (34.0)	30 (40.5)	16 (41.0)	1 (20.0)	19 (31.7)	
Ever smoker	215 (66.2)	97 (66.0)	44 (59.5)	23 (59.0)	4 (80.0)	41 (68.3)	
**Pleural fluid**							
RBC (/μL)	4,270 (1,200–22,100)	5,250 (2,440–25,650)	8,400 (1,250–72,375)	3,100 (2,600–8,000)	5,400 (1,075–31,650)	1,600 (640–4,800)	0.001
WBC (/μL)	1,157 (440–2,845)	1,358 (539–2,785)	3,690 (1,021–12,335)	2,050 (1,300–2,868)	793 (345–1,438)	440 (190–900)	<0.001
Neutrophil (%)	15.5 (3.0–43.0)	9.0 (2.0–26.8)	76.0 (55.0–87.8)	7.0 (1.0–9.0)	7.0 (2.0–24.3)	11.0 (4.5–27.5)	<0.001
Lymphocyte (%)	46.0 (17.0–72.3)	47.5 (18.3–72.8)	8.0 (2.3–17.0)	75.0 (66.0–83.0)	61.5 (39.3–79.0)	47.0 (26.5–72.0)	<0.001
Histiocyte (%)	16.0 (9.0–25.2)	17.0 (11.0–31.5)	10.5 (6.0–18.5)	13.0 (7.0–22.0)	17.0 (12.8–31.0)	17.0 (10.5–25.0)	0.003
Protein (g/dL)	3.6 (2.7–4.6)	4.2 (3.3–4.8)	3.4 (2.8–4.3)	4.9 (4.2–5.6)	3.9 (3.2–4.7)	2.0 (1.7–2.6)	<0.001
Albumin (g/dL)	2.1 (1.7–2.7)	2.6 (2.1–3.0)	1.8 (1.7–2.4)	2.9 (2.6–2.9)	2.1 (1.6-2.6)	1.6 (1.6–1.7)	<0.001
Amylase (IU/L)	26.8 (17.7–38.8)	32.0 (23.6–57.5)	21.9 (14.6–28.5)	37.9 (33.8–45.7)	28.4 (18.9-38.0)	18.8 (13.1–29.2)	<0.001

Variable	Total (N=325)	Exudate (n=265)				Transudate (n=60)	*p value**
		MPE (n=147)	PPE (n=74)	TB pleurisy (n=39)	Others (n=5)		
Glucose (mg/dL)	112 (83–139)	105 (88–137)	90.8 (12.8–129.4)	118 (86–146)	115 (99-135)	129 (106–157)	<0.001
LDH (IU/L)	224.3 (141.0–505.6)	288.7 (198.9–642.2)	589.6 (221.6–1,261.1)	294.0 (167.5–476.3)	171.8 (146.7–287.7)	93.8 (73.8–141.3)	<0.001
ADA (IU/L)	17.6 (11.1–28.0)	19.5 (11.5–24.7)	24.2 (14.0–36.7)	46.5 (31.9–75.6)	15.3 (12.3–23.8)	9.3 (6.8–14.0)	<0.001
**Serum**							
Protein (g/dL)	6.4 (5.8–7.0)	6.5 (6.1–7.0)	6.1 (5.7–6.7)	7.0 (6.4–7.2)	6.0 (5.5–6.9)	6.0 (5.1–6.7)	0.001
Albumin (g/dL)	3.6 (3.2–3.9)	3.6 (3.4–3.9)	3.5 (3.2–3.8)	3.9 (3.7–4.0)	3.4 (3.2–3.9)	3.3 (2.9–3.7)	0.002
LDH (IU/L)	205.0 (165.5–272.3)	209.5 (180.5–283.0)	182.0 (142.3–234.3)	198.0 (163.0–223.0)	218.5 (185.8–319.8)	207.0 (169.0–276.0)	0.082

Data are presented as median (interquartile range) for continuous variables or n (%) for categorical variables. ADA = adenosine deaminase; LDH = lactate dehydrogenase; MPE = malignant pleural effusion; PPE = parapneumonic effusion; RBC = red blood cell; TB = tuberculous; RBC = red blood cell; WBC = white blood cell.

* P values were calculated for comparison among the four main etiologic groups (MPE, PPE, TB pleurisy, and transudate); the "Others" group (n = 5) was excluded from statistical testing due to the small sample size.

Supplementary Table S2. Markers for immunohistochemistry according to various tumors

Tumor Type	IHC Markers
Lung cancer	TTF-1, Napsin A, p63, chromogranin, synaptophycin, CD56, PD-L1
Breast cancer	ER, GATA3
Ovarian cancer	PAX8, WT1, ER
Gastric cancer	CDX2, PD-L1
Lymphoma	CD3, CD20, CD45

CD3 = cluster of differentiation 3; CD20 = cluster of differentiation 20; CD45 = cluster of differentiation 45; CD56 = cluster of differentiation 56; CDX2 = caudal type homeobox 2; ER = estrogen receptor; GATA3 = GATA binding protein 3; IHC = immunohistochemistry; PAX8 = paired box gene 8; PD-L1 = programmed death-ligand 1; TTF-1 = thyroid transcription factor 1; WT1 = Wilms tumor 1

Supplementary Table S3. Conditional and population-level diagnostic sensitivity of cytology, pleural tissue debris, and their combination for the diagnosis of malignant pleural effusion

Diagnostic modality	Positive cases	Negative cases	Sensitivity (%)	95% CI*
**Conditional (specimen-level) sensitivity (denominator, patients with detectable tissue debris, n=95)**				
Cytology with cell block	63	32	66.3	55.9 – 75.7

Diagnostic modality	Positive cases	Negative cases	Sensitivity (%)	95% CI*
Pleural tissue debris	74	21	77.9	68.2 – 85.8
Cytology + Tissue debris (either positive)	80	15	84.2	75.3 – 90.9
**Population-level (intention-to-diagnose) sensitivity (denominator, all patients with MPE, n=147)**				
Cytology with cell block	103	44	70.1	62.0 – 77.3
Pleural tissue debris	74	73	50.3	42.0 – 58.7
Cytology + Tissue debris (either positive)	120	27	81.6	74.4 – 87.5

*95% confidence intervals were calculated using the exact (Clopper–Pearson) method.

CI = confidence interval; MPE = malignant pleural effusion.

Supplementary Table S4. Statistical comparison of paired diagnostic sensitivities (McNemar's test and Cochran's Q test)

Comparison	Discordant pairs* (b, c)	Statistical test	p value
**Pairwise comparisons (McNemar's test, exact binomial)**			
Tissue debris vs Cytology	(17, 6)	McNemar's exact	0.035
Combined vs Cytology	(17, 0)	McNemar's exact	< 0.001
Combined vs Tissue debris	(6, 0)	McNemar's exact	0.031
**Overall comparison of three paired proportions**			

Comparison	Discordant pairs* (b, c)	Statistical test	p value
Cytology vs Tissue debris vs Combined	—	Cochran's Q (Q = 19.39, df = 2)	< 0.001

*Discordant pairs are denoted (b, c), where **b** = positive by the first modality and negative by the second, and **c** = the reverse. For the combined-vs-single comparisons, c = 0 because the combined definition is a superset of each single modality.

df = degrees of freedom; Q = Cochran's Q statistic.

Supplementary Table S5. Adequacy of pleural tissue debris for molecular testing according to assay platform in patients with non-small cell lung cancer (n=58)

Assay platform	Tests performed	Reportable results	Adequacy (%)	95% CI*
PCR-based assay	58	40	69.0	55.5 – 80.5
Targeted NGS panel	58	35	60.3	46.6 – 73.0
At least one assay (per patient)**	58	40	69.0	55.5 – 80.5

*95% confidence intervals were calculated using the exact (Clopper–Pearson) method.

**Both assays yielded reportable results in 35 patients (60.3%), PCR alone in 5 (8.6%), NGS alone in none, and neither assay in 18 (31.0%). The difference in adequacy between the two platforms did not reach statistical significance (McNemar's exact test, p = 0.063).

CI = confidence interval; NGS = next-generation sequencing; PCR = polymerase chain reaction.
